# Sentinel lymph node biopsy in patients with ductal carcinoma *in situ*: systematic review and meta-analysis

**DOI:** 10.1093/bjsopen/zrac022

**Published:** 2022-04-05

**Authors:** Matthew G. Davey, Colm O’Flaherty, Eoin F. Cleere, Aoife Nohilly, James Phelan, Evan Ronane, Aoife J. Lowery, Michael J. Kerin

**Affiliations:** Department of Surgery, The Lambe Institute for Translational Research, National University of Ireland, Galway, Ireland

## Abstract

**Background:**

Axillary lymph node status remains the most powerful prognostic indicator in invasive breast cancer. Ductal carcinoma *in situ* (DCIS) is a non-invasive disease and does not spread to axillary lymph nodes. The presence of an invasive component to DCIS mandates nodal evaluation through sentinel lymph node biopsy (SLNB). Quantification of the necessity of upfront SLNB for DCIS requires investigation. The aim was to establish the likelihood of having a positive SLNB (SLNB+) for DCIS and to establish parameters predictive of SLNB+.

**Methods:**

A systematic review was performed as per the PRISMA guidelines. Prospective studies only were included. Characteristics predictive of SLNB+ were expressed as dichotomous variables and pooled as odds ratios (o.r.) and associated 95 per cent confidence intervals (c.i.) using the Mantel–Haenszel method.

**Results:**

Overall, 16 studies including 4388 patients were included (mean patient age 54.8 (range 24 to 92) years). Of these, 72.5 per cent of patients underwent SLNB (3156 of 4356 patients) and 4.9 per cent had SLNB+ (153 of 3153 patients). The likelihood of having SLNB+ for DCIS was less than 1 per cent (o.r. <0.01, 95 per cent c.i. 0.00 to 0.01; *P* < 0.001, *I*^2^ = 93 per cent). Palpable DCIS (o.r. 2.01, 95 per cent c.i. 0.64 to 6.24; *P* = 0.230, *I*^2^ = 0 per cent), tumour necrosis (o.r. 3.84, 95 per cent c.i. 0.85 to 17.44; *P* = 0.080, *I*^2^ = 83 per cent), and grade 3 DCIS (o.r. 1.34, 95 per cent c.i. 0.80 to 2.23; *P* = 0.270, *I*^2^ = 0 per cent) all trended towards significance in predicting SLNB+.

**Conclusion:**

While aggressive clinicopathological parameters may guide SLNB for patients with DCIS, the absolute and relative risk of SLNB+ for DCIS is less than 5 per cent and 1 per cent, respectively. Well-designed randomized controlled trials are required to establish fully the necessity of SLNB for patients diagnosed with DCIS.

**Registration number:**

CRD42021284194 (https://www.crd.york.ac.uk/prospero/)

## Teaser

This meta-analysis evaluates the clinical utility of routine sentinel lymph node biopsy for surgery in the cases of ductal carcinoma *in situ*. The results from this analysis support the de-escalation of sentinel lymph node biopsy in such cases, unless directed otherwise by aggressive clinicopathological data, such as tumour necrosis and palpable disease.

## Introduction

Following the widespread establishment and implementation of population-based breast cancer screening programmes and digitalized imaging, detection rates of ductal carcinoma *in situ* (DCIS) have increased dramatically^1,2^, with DCIS now constituting 20 to 25 per cent of all breast cancers^3^. DCIS is a premalignant precursor disease to invasive ductal carcinoma (IDC), which is characterized by abnormal proliferation of epithelial cells confined within the basal membrane of breast glandular tissue^4^. Theoretically, DCIS is non-invasive, and therefore does not possess any metastatic potential for locoregional spread to axillary lymph nodes. Therefore, routine lymph node sampling to stage the axilla in the setting of DCIS is unnecessary^5^.

Axillary lymph node status remains the most powerful prognostic indicator in patients diagnosed with breast cancer^6^. Therefore, sentinel lymph node biopsy (SLNB) is currently mandated in all cases suspected to be invasive breast cancer. In patients with clinically node-negative invasive disease, SLNB is performed and provides non-inferior survival outcomes to axillary lymph node dissection (ALND)^7–9^. The ACOSOG Z0011 trial demonstrated that patients with invasive breast cancer with limited metastatic disease in the axilla may be spared ALND^10^. These trials have evolved the paradigm for patients with invasive disease; however, there has been no randomized controlled trial (RCT) published to date investigating the value of performing routine SLNB for patients with DCIS.

At present, a SLNB is only performed in select cases of DCIS, such as cases with large volumes of disease requiring mastectomy, when there is an anticipated risk of upstaging to invasive disease on the specimen following histopathological evaluation. Best practice guidelines, such as those reported by American Society of Clinical Oncology and National Institute for Health and Care Excellence, support SLNB in cases requiring mastectomy, in cases of extensive DCIS (greater than 50 mm), or those with clinical or radiological evidence suggestive of possible invasive disease^11,12^. However, the evidence supporting such recommendations may be challenged owing the sparsity of data supporting formal staging of the axilla, as well as the absence of concise selection criteria^5,13^. Thus, it is reasonable to suggest that there is a proportion of patients being treated for DCIS who currently undergo unnecessary upfront SLNB. Moreover, histopathological evaluation of the resected breast specimen is mandatory, which will ultimately dictate the indication for SLNB based on the presence of invasive cancer. Therefore, the rationale for performing upfront SLNB as routine for patients being treated for DCIS should be challenged. Accordingly, the aim of the current systematic review and meta-analysis was to establish the likelihood of having lymph node metastases on SLNB (SLNB+) in patients being treated surgically for DCIS, and to establish clinicopathological parameters that may be useful in predicting those likely to be SLNB+ at the time of breast surgery for DCIS.

## Methods

A systematic review was conducted in accordance to the PRISMA checklist^14^ and Meta-analysis Of Observational Studies in Epidemiology (MOOSE) guidelines^15^. Given the nature of this review, local institutional ethical approval was not required. The study was registered in PROSPERO (CRD42021284194).

### Population, intervention, comparison, outcome (PICO) tool

Using the PICO framework^16^, the aspects the authors wished to address were:

- Population—female patients, aged 18 years or older, with newly diagnosed DCIS breast cancer, with histologically or radiologically confirmed DCIS in the preoperative setting.
- Intervention—any patient in the selected group who underwent staging with SLNB and were subsequently found to have positive disease in the axilla at the time of their breast cancer surgery for DCIS.
- Comparison—any patient in the selected group who underwent staging with SLNB and were subsequently found not to have positive disease in the axilla at the time of their breast cancer surgery for DCIS.
- Outcomes—primary outcomes included SLNB+ (including micro- and/or macrometastatic disease in the axillary lymph nodes) following an initial diagnosis of DCIS. Secondary outcomes included any clinicopathological features predictive of those likely to have SLNB+ following an initial diagnosis of DCIS.

### Search strategy

An electronic search was performed of the PubMed, Embase, and Scopus databases on 25 May 2021 for relevant studies that would be suitable for inclusion in this study. The search was performed of all fields under the following headings: (ductal carcinoma in situ), (sentinel lymph node biopsy), which were linked by the Boolean operator ‘AND’. Included studies were limited to those published in the English language and studies were not restricted based on year of publication. For retrieved studies, their titles were initially screened, before the abstracts and full texts which were deemed appropriate were reviewed.

### Inclusion and exclusion criteria

Studies meeting the following inclusion criteria were included: studies assessing patients with histologically confirmed DCIS breast cancer in the breast preoperatively with or without positive disease in the axilla (assessed using SLNB); and studies had to include data that were collected prospectively (included studies did not necessarily have to be controlled) and included prospectively collected registry data. Studies meeting any of the following exclusion criteria were excluded: studies with patients with histologically confirmed invasive breast cancer (e.g. IDC histological subtype); studies including data that were not collected prospectively; review articles; studies including fewer than five patients in their series or case reports; or editorial articles.

### Data extraction and quality assessment

The literature search was performed by two independent reviewers (M.G.D and C.’OF.) using a predesigned search strategy, which had been developed under the supervision of the senior author (M.J.K.). Duplicate studies were manually removed. Each reviewer then reviewed the titles, abstracts, and/or full texts of the retrieved manuscripts to ensure all inclusion criteria were met, before extracting the following data: first author name; year of publication; study design; level of evidence; study title; number of patients; number of patients who underwent SLNB; number of patients who underwent SLNB and subsequently had axillary lymph nodes positive for cancer cells; clinicopathological and surgical parameters of the entire patient population; and clinicopathological and surgical parameters of the entire patient population with positive axillary lymph nodes. The definition of SLNB+ included ‘micro- and macrometastases only’ in accordance with the AJCC version 8 guidelines for breast cancer^17^. This definition excluded isolated tumour cells (ITCs). Risk of bias and methodology quality assessment was performed in accordance with the Risk Of Bias In Non-randomised Studies of Interventions (ROBINS-I)^18,19^. In case of discrepancies in opinion between the reviewers, a third reviewer (E.F.C) was asked to arbitrate.

### Statistical analysis

Clinicopathological and treatment characteristics were expressed as descriptive statistics with Fisher’s exact and χ^2^ tests, as appropriate^20^, to determine clinicopathological features associated with SLNB+. Determining the likelihood of SLNB+ in cases of DCIS with SLNB and relevance of clinicopathological parameters predictive of SLNB+ were assessed as dichotomous variables, expressed as odds ratios (o.r.) with corresponding 95 per cent confidence intervals (c.i.) using the Mantel–Haenszel method. Either fixed- or random-effects models were applied on the basis of whether significant heterogeneity (*I*^2^ > 50 per cent) existed between studies included in the analysis. Symmetry of funnel plots was used to assess publication bias. Statistical heterogeneity was determined using *I*^2^ statistics. Statistical significance was determined to be *P* < 0.050. Statistical analysis was performed using Review Manager (RevMan), Version 5.4 (Nordic Cochrane Centre, Copenhagen, Denmark).

## Results

### Literature search

**Fig. 1:**
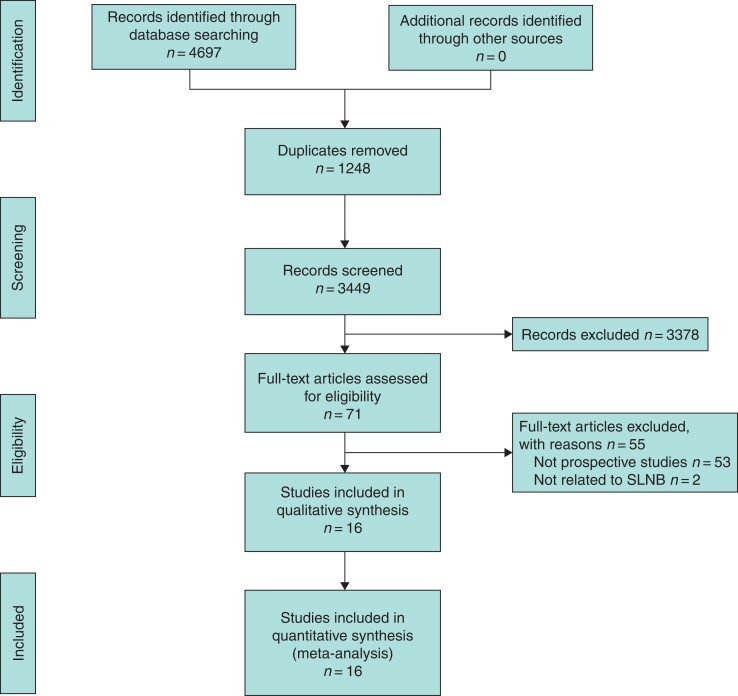
PRISMA flow diagram detailing the systematic search process

The initial electronic search retrieved 4697 studies. Following removal of the 1248 identified duplicate studies, the remaining 3449 titles were screened for relevance, of which 576 had their abstracts and 71 had their full texts assessed for eligibility. Overall, 16 prospective studies fulfilled the inclusion criteria and were subsequently included in this systematic review^21–36^, as outlined in *Fig. 1*. Details of individual included studies are outlined in *Table 1*.

SLNB, sentinel lymph node biopsy.

**Table 1:** Details of the 16 prospective studies in this systematic review and meta-analysis

Author	Year	Country	*n*	Mean patient age (years)	Age range (years)	ROBINS-I
**Kelly**	2003	USA	420	54.3	—	2
**Mittendorf**	2005	USA	85	57.0	29–85	2
**Guillot**	2014	France	241	51.0	28–82	2
**Goyal**	2006	UK	367	58.0	49–81	2
**Moran**	2007	ROI	62	—	50–65	2
**Usmani**	2011	Kuwait	23	50.0	37–78	3
**Zetterlund**	2014	Sweden	1273	60.0	26–92	2
**D’Eredita**	2009	Italy	90	56.0	27–86	3
**Collado**	2010	Spain	65	51.9	38–69	3
**Klauber-DeMore**	2000	USA	76		—	3
**Fancellu**	2012	Italy	140	56.0	26–89	2
**Intra**	2003	Italy	854	—	—	2
**Tunon-de-Lara**	2015	France	227	—	24–83	2
**van la Parra**	2008	Netherlands	51	59.0	39–81	3
**Leidenius**	2006	Finland	74	56.0	38–91	3
**Park**	2013	ROK	340	48.5	25–78	3

ROBINS-I, risk of bias in non-randomised studies of interventions; ROI, Republic of Ireland; ROK, Republic of Korea.

### Study characteristics

In total, 4388 patients diagnosed with DCIS were included in this study. Mean patient age at diagnosis was 54.8 (range 24 to 92) years. Overall, 67.6 per cent of patients underwent mastectomy (2514 of 3719) and 32.4 per cent underwent breast conservation surgery (1205 of 3719; 13 studies). In total, 72.5 per cent of patients underwent SLNB (3156 of 4356) and 4.9 per cent had SLNB+ (153 of 3153). Of the 4388 patients included in this study, 314 had invasive cancer in the breast present on their final histology (7.2 per cent). Of these, 26.8 per cent had SLNB+ (84 of 314). Pooled clinicopathological and treatment data from the 16 included studies are outlined in *Table 2*.

**Table 2:** Clinicopathological and treatment characteristics of the included patients in this study

Parameter	Total group	SLNB+ group	*P* *
**Screening detected**	364	11	0.350
**Symptomatic (palpable)**	96	5	
**Necrosis present**	691	30	<0.001
**Necrosis absent**	1127	16	
**Microcalcification present**	226	13	0.309
**Microcalcification absent**	126	15	
**Grade 1**	325	7	0.969†
**Grade 2**	1039	24	
**Grade 3**	1614	35	
**Grade 1/2**	1727	31	0.447
**Grade 3**	1614	35	
**ER+**	825	9	0.299
**ER−**	381	7	
**PgR+**	802	9	0.386
**PgR−**	403	7	
**HER2+**	479	4	0.270
**HER2−**	765	12	
**Ki67 < 20%**	324	9	0.096
**Ki67 > 20%**	446	5	
**BCS**	1205	12	0.016
**Mastectomy**	2514	9	

SLNB+, metastatic lymph nodes on sentinel lymph node biopsy; ER, oestrogen receptor; PgR, progesterone receptor; HER2, human epidermal growth factor receptor-2; BCS, breast conservation surgery. *P values from Fisher’s exact test unless otherwise stated; †χ^2^ test.

### Axillary lymph node positivity

**Fig. 2:**
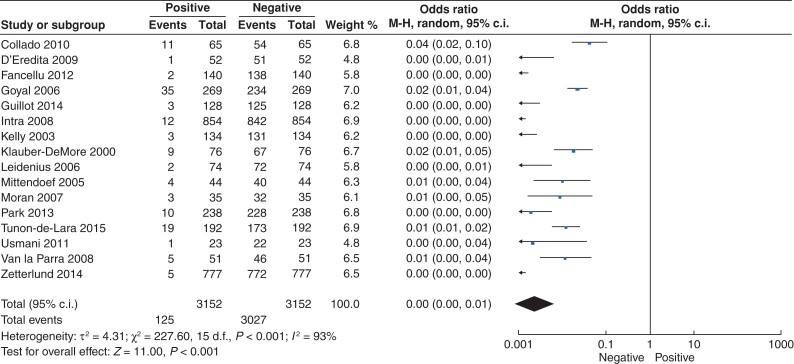
Forest plot illustrating the likelihood of having metastatic disease in axillary lymph nodes in patients with ductal carcinoma in situ

As previously outlined, 4.9 per cent of the 3153 patients who underwent SLNB had positive disease on their SLNB (153 of 3153). Of those reporting type of metastases, 58.4 per cent had micrometastases present on SLNB (66 of 113), while 41.6 per cent had macrometastatic disease present on SLNB (47 of 113). For the 3153 patients undergoing SLNB, the likelihood of having SLNB+ was less than 1 per cent (o.r. < 0.01, 95 per cent c.i. 0.00 to 0.01; *P* < 0.001, *I*^2^ = 93 per cent) (*Fig. 2*).

Of note, ITCs were present in 0.8 per cent of cases (26 of 3153). Overall, 4.7 per cent of patients proceeded to axillary lymph node dissection (148 of 3153). Details in relation to axillary lymph node status are provided in *Table 3*.

**Table 3:** Details in relation to sentinel lymph node biopsies, lymph node status, and axillary lymph node dissection

Parameter	*n* (%)
**Underwent SLNB**	3156 (79.6)
**Did not undergo SLNB**	1200 (20.4)
**SLNB−**	3000 (95.1)
**SLNB+**	153 (4.9)
**Not reported**	3 (<0.1)
**Micrometastases**	66 (43.1)
**Macrometastases**	47 (30.7)
**Not reported**	40 (26.1)
**ITCs**	26 (0.8)
**ALND**	148 (4.7)

SLNB, sentinel lymph node biopsy; ITCs, isolated tumour cells; ALND, axillary lymph node dissection.

### Clinicopathological predictors of axillary lymph node positivity

**Fig. 3:**
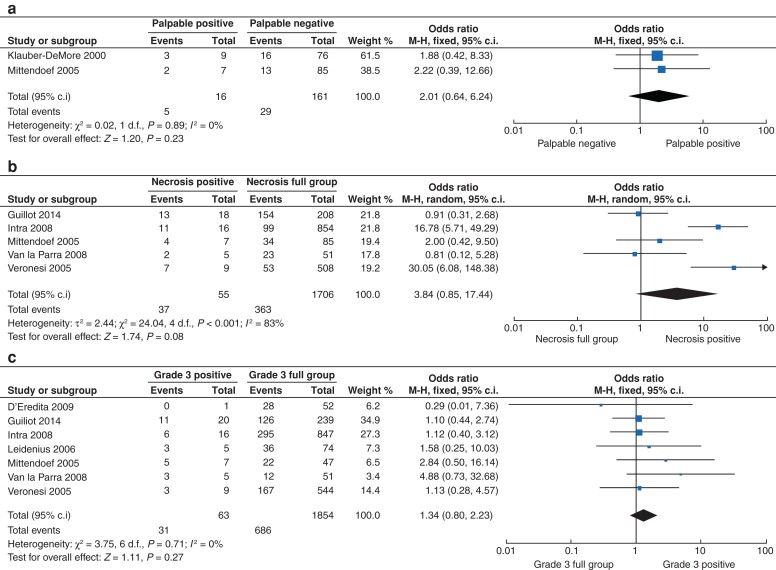
Forest plot illustrating the ability of **a** palpable disease, **b** tumour necrosis, and **c** grade 3 ductal carcinoma *in situ* in predicting metastatic disease in axillary lymph nodes.

The presence of tumour necrosis (*P* < 0.001) and undergoing mastectomy (*P* = 0.016) were both associated with having SLNB+ for DCIS surgery (*Table 2*). Being symptomatic (or having palpable DCIS (o.r. 2.01, 95 per cent c.i. 0.64 to 6.24; *P* = 0.230, *I*^2^ = 0 per cent)) (*Fig. 3a*), the presence of tumour necrosis (o.r. 3.84, 95 per cent c.i. 0.85 to 17.44; *P* = 0.080, *I*^2^ = 83 per cent) (*Fig. 3b*), and the presence of grade 3 disease (o.r. 1.34, 95 per cent c.i. 0.80 to 2.23; *P* = 0.270, *I*^2^ = 0 per cent) (*Fig. 3c*) all trended towards significance in predicting patients likely to have SLNB+. Forest plots for other clinicopathological parameters and predicted value for SLNB+ are outlined in *Fig. S1*.

## Discussion

This systematic review and meta-analysis assessed the value of performing routine SLNB in patients being treated surgically for DCIS. For decades, the surgical conundrum surrounding the appropriateness of SLNB for cases of DCIS has been debated by surgical oncologists, owing to a lack of clear consensus. The results of the current meta-analysis were derived from the highest level of evidence available (prospectively collected data only). Similarly to the work of El Hage Chehade *et al.*^37^, the overall absolute likelihood of capturing metastatic disease in axillary lymph nodes following SLNB was approximately 5 per cent, with an estimated relative detection rate of less than 1 per cent. Although this illustrates there is the potential to detect metastatic disease in the axilla at SLNB, the data do not support the performance of *a priori* lymph node sampling as routine in all cases of DCIS. Therefore, the clear message from this meta-analysis is that the surgical oncologist, at their own discretion, should avoid performing SLNB for DCIS surgery, unless there is high suspicion for invasive disease.

In this analysis, 72.5 per cent of patients underwent SLNB for DCIS, yet less than 5 per cent of these had SLNB+. This suggests that there is a tendency for breast surgeons to stage the axilla in cases of DCIS, despite acknowledgement that this is a non-invasive disease^38^. Debate fuelling the controversy of sentinel node mapping as routine management of DCIS is based on the following fundamental concepts. Primarily, the resecting surgeon is aware that there is a proportion of patients with DCIS who will ultimately progress to develop IDC^39,40^. Additionally, sentinel lymph node status remains the most crucial predictor of prognosis in invasive carcinoma^6^, and if invasive disease is detected, axillary staging is fundamental to therapeutic decision-making in the adjuvant setting^41,42^. These principles remain at the crux of the argument supporting routine SLNB for DCIS. Nevertheless, the real-world data presented in the current analysis highlight that there is a less than a 5 per cent absolute risk of invasive cancer being detected on SLNB on final histology. Therefore, judicious use of SLNB is required within the setting of DCIS, with limited exceptions.

This study may be challenged by being perceived as oversimplifying the requirement for routine axillary staging in cases of DCIS. However, these data illustrate that there are certain clinicopathological parameters associated with SLNB+, which may be useful in guiding preoperative decision-making in relation to SLNB. These data suggest that having palpable disease (o.r. 2.01), the presence of tumour necrosis (o.r. 3.84), and having grade 3 DCIS (o.r. 1.34) are useful tumour characteristics for predicting SLNB+. This is somewhat unsurprising. Palpable DCIS has been associated with aggressive clinicopathological features, such as high-grade and comedo necrosis^43^, as well as invasive cancer in approximately 25 per cent of cases^44^. Therefore, it is fair to expect that such cases may require mastectomy, particularly when palpable DCIS (or large-volume DCIS, which will require mastectomy) is a reasonable parameter for which SLNB may be considered. Furthermore, Kerlikowske *et al.* reported that palpable DCIS, combined with high-grade histology, independently predicts DCIS recurrence as invasive disease^45^. High-grade DCIS (or grade 3 DCIS) shows large-sized, pleomorphic neoplastic cells, with large and irregularly shaped nuclei, with multiple, prominent nucleoli and high mitotic indices, indicating high proliferative potential^46^. Moreover, these cancers often show a necrotic core^46^, and recent prospective data from the Sloane Project illustrated that high-grade DCIS correlated with poorer oncological outcome than those with low–intermediate grade DCIS after more than 9 years of follow-up^47^. Additionally, comedo necrosis (or central necrosis) occurs in highly proliferative cancers that outgrow their supply of nutrients and oxygen, causing deprivation and tumour apoptosis^48^. Unsurprisingly, comedo necrosis has been correlated with aggressive tumour features such as increased tumour burden, higher proliferative potential, and poorer anticipated prognosis^49^, with strong associations with ipsilateral invasive cancer recurrence^50^. This suggests that caution is required when deciding on the appropriate staging of such cases. It is acknowledged that grade and necrosis are contemporary characteristics in the College of American Pathologists reporting protocol for the histopathological specimens of DCIS^51^, which further emphasizes their importance in cases of DCIS. Therefore, when the breast multidisciplinary team meeting is faced with a case of palpable, grade 3, necrosing DCIS, consideration for SLNB is justified, to ensure adequate staging of the axilla in the incidence that the resected tumour is upstaged to invasive disease on final histology.

While the era of molecular profiling and minimally invasive surgery have revolutionized the approach to the management of invasive breast cancer^7,8,10,41,52–54^, the translational research efforts to progress the management of DCIS have lagged behind considerably. For example, multigene assays, such as the 21-gene and 70-gene signatures, have become embedded into the paradigm for certain early-stage invasive cancers^41,53,55–57^. In contrast, the uptake of the clinically validated 12-gene DCIS recurrence assay has been less successful^58,59^. With respect to surgical management of the axilla in cases of DCIS, there is currently just one ongoing clinical trial focused on enhancing surgical practice for patients with DCIS: the SentiNot 2.0 trial (NCT04722692) is currently randomizing patients to either radioisotope (control) or superparamagnetic iron oxide (SPIO) tracing of the axillary nodes at delayed SLNB in patients with a preoperative diagnosis of DCIS, who are subsequently found to have invasive disease on final histology^60^. Similar to the message of the current meta-analysis, SentiNot 2.0 proposes a delay in performing SLNB in patients undergoing mastectomy for DCIS. Therefore, the next generation of prospective trials should look to evaluate the necessity of upfront SLNB for DCIS, in order to provide clear consensus to the debate regarding the most appropriate management of the axilla in such circumstances.

This meta-analysis is subject to several limitations. In the absence of well-designed RCTs evaluating the necessity of SLNB in DCIS surgery, cautious interpretation of these results is required. Observational studies of a non-randomized design, in particular those where retrospective analysis of prospectively collected data is performed, are subject to the inherent risk of selection and confounding biases. In this study, surgical procedures performed in those with SLNB+ were outlined in just 13.7 per cent of cases (21 of 153), meaning the full necessity of SLNB in cases requiring mastectomy for DCIS has not been fully evaluated. In such circumstances, axillary staging may be appropriate at the time of resection^12^, as small invasive cancers are occasionally present on final histology. Detecting clinicopathological characteristics predictive of SLNB+ was the secondary outcome in this study; however, the paucity of such data may bring the validity of these results into question (as outlined in *Table 2*). Despite these limitations, this meta-analysis provides the highest quality of available prospective data reflecting current management strategies of the axilla in cases of DCIS.

This systematic review and meta-analysis illustrates an absolute likelihood of less than 5 per cent of having metastatic disease following SLNB for DCIS, with an estimated relative risk of less than 1 per cent. It therefore suggests that there is limited premise for upfront axillary lymph node sampling in the setting of DCIS. However, aggressive clinicopathological characteristics, such as having a clinically palpable tumour, or possessing comedo necrosis and/or high-grade DCIS on diagnostic core biopsy, may be useful to guide preoperative decision-making as to when SLNB may be required. The provision of well-designed prospective studies are essential to evaluate properly the de-escalation of upfront SLNB in patients being treated surgically for DCIS.

## Supplementary Material

zrac022_Supplementary_Data

## Data Availability

Data are available upon request at the discretion of the corresponding author.
