# Supplementary material for: Sentinel lymph node biopsy in patients with ductal carcinoma in situ: systematic review and meta-analysis
Source: BJS Open. 2022 Apr 5;6(2):zrac022. doi: 10.1093/bjsopen/zrac022 (PMC8982203; doi:10.1093/bjsopen/zrac022)
Supplement: zrac022_Supplementary_Data [file zrac022_supplementary_data.zip › Supplementary_Figure_1.docx]

**Figure S1.** Forest plot illustrating the ability of (A) Ki-67 proliferation indices, (B) microcalcifications and (C) surgery in predicting metastatic disease in axillary lymph nodes.


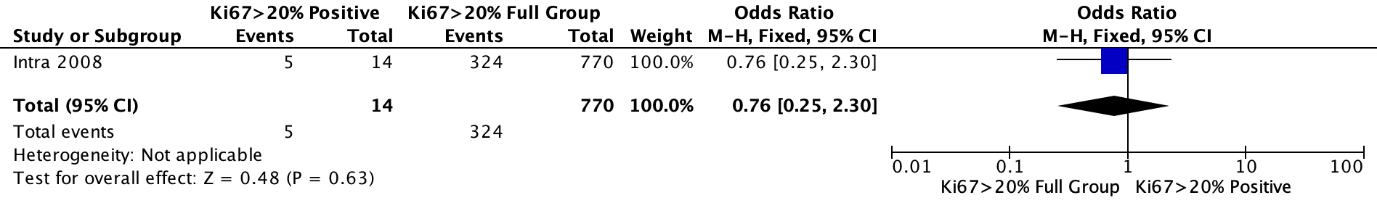


A

B


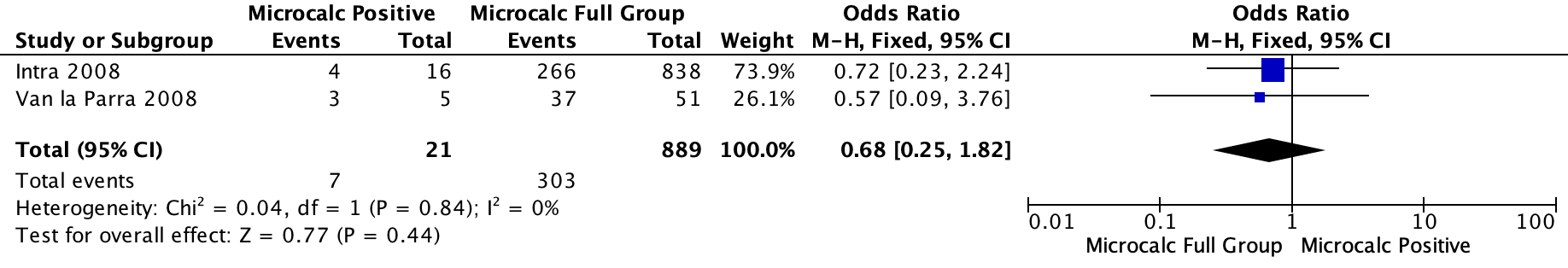


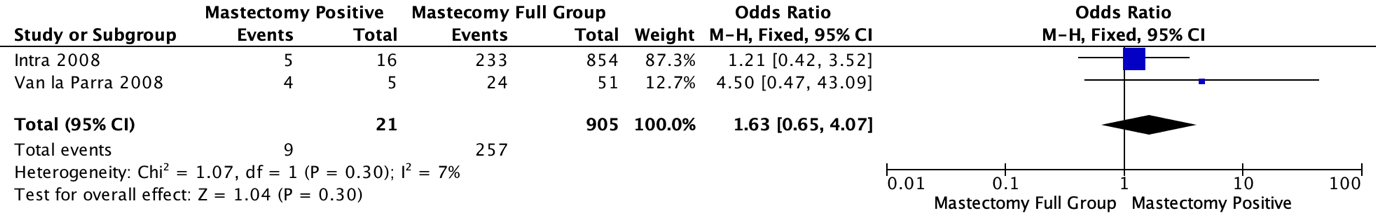


C
